# Targeting YAP-dependent MDSC infiltration impairs tumor progression

**DOI:** 10.1186/2051-1426-3-S2-P421

**Published:** 2015-11-04

**Authors:** Guocan Wang, Xin Lu, Prasenjit Dey, Pingna Deng, Chia Chin Wu, Shan Jiang, Zhuangna Fang, Kun Zhao, Ramakrishna Konaparthi, Sujun Hua, Jianhua Zhang, Elsa M Li Ning Tapia, Avnish Kapoor, Neelay Patel, Zhenglin Guo, Vandhana Ramamoorthy, Trang Tieu, Tim Heffernan, Di Zhao, Xiaoying Shang, Sunada Khadka, Pingping Hou, Baoli Hu, Qing Chang, Patricia Troncoso, Christopher Logothetis, Mark McArthur, Lynda Chin, Y Alan Wang, Ronald DePinho

**Affiliations:** 1 The University of Texas MD Anderson Cancer Center, Houston, TX, USA; 2 Md anderson cancer center, Houston, TX, USA; 3 UT MD Anderson Cancer Center, Houston, TX, USA

## Background

Myeloid-derived suppressor cells (MDSCs) represent a phenotypically heterogeneous population of immature myeloid cells that play a tumor-promoting role by maintaining a state of immunological anergy and tolerance. Similar to other solid tumor types, Prostate cancer (PCa) is characterized by a rich tumor-stroma interaction network that forms the TME. MDSC abundance in the blood correlates with circulating Prostate Specific Antigen (PSA) levels in PCa patients. MDSCs have been identified recently as a TME constituent in an indolent PCa mouse model with conditional *Pten* deletion, which antagonized senescence during early tumorigenesis. However, the molecular signaling mechanisms and their cellular origins underlying the recruitment of MDSCs are not well understood and the extent to which MDSCs facilitate PCa progression has not been determined.

## Results

The signaling mechanisms between cancer cells and infiltrating immune cells of prostate cancer may illuminate novel therapeutic approaches. Here, utilizing a murine prostate adenocarcinoma model driven by loss of Pten and Smad4 (Pb-Cre; Pten^L/L^;Smad4 ^L/L^), we identify polymorphonuclear myeloid-derived suppressor cells (MDSCs) as the major infiltrating immune cell type and depletion of MDSCs blocks progression. Employing a dual cancer cell and host cell reporter prostate cancer model system (Pb-Cre; Pten^L/L^;Smad4 ^L/L^;Rosa26-mTmG^L/+^), transcriptomic profiling of epithelial and stromal constituents identified Cxcl5 as a cancer-secreted chemokine to attract Cxcr2-expressing MDSCs and, correspondingly, pharmacological inhibition of Cxcr2 impeded tumor progression. Integrated analyses identified hyperactivated Hippo-YAP signaling in driving Cxcl5 upregulation in cancer cells through YAP-TEAD complex and promoting MDSCs recruitment. Clinico-pathological studies reveal upregulation and activation of YAP1 in a subset of human prostate tumors, and the YAP1 signature is enriched in these primary prostate tumor samples with stronger expression of MDSC relevant genes. Together, YAP-driven MDSC recruitment via heterotypic Cxcl5-Cxcr2 signaling, which promotes prostate tumor progression, reveals effective anti-MDSC therapeutic interventions for advanced prostate cancer.

## Significance

This study employs a novel autochthonous prostate cancer model to demonstrate a critical role of MDSCs in tumor progression. In addition, we discover a cancer cell non-autonomous function of Hippo-YAP pathway in regulation of Cxcl5, a ligand for Cxcr2 expressing MDSCs. Pharmacologic elimination of MDSCs or blocking the heterotypic CxCl5-Cxcr2 signaling circuit elicits robust anti-tumor responses in vivo and prolongs survival.

